# Hepatitis C virus fails to activate NF-kappaB signaling in plasmacytoid dendritic cells

**DOI:** 10.1186/1742-4690-9-S1-O5

**Published:** 2012-05-25

**Authors:** Ruzena Stranska, Jonathan Florentin, Clélia Dental, Besma Aouar, Francoise Gondois-Rey, David Durantel, Thomas F Baumert, Jacques A Nunes, Daniel Olive, Ivan Hirsch

**Affiliations:** 1 Centre de Recherche en Cancérologie de Marseille, Marseille, France

## Introduction

Plasmacytoid dendritic cells (pDCs) respond to viral infection by production of interferon α (IFN-α), proinflammatory cytokines and cell differentiation. The elimination of hepatitis C virus (HCV) in more than 50% of chronically infected patients by treatment with IFN-α suggests that pDCs can play an important role in the control of HCV infection. pDCs exposed to HCV-infected hepatoma cells, in contrast to cell-free HCV virions, produce large amounts of IFN-α.

## Materials and methods

To further investigate the molecular mechanism of HCV sensing, we studied whether exposure of pDCs to HCV-infected hepatoma cells activates in parallel to interferon regulatory factor 7 (IRF7)-mediated production of IFN-α also nuclear factor kappa B (NF-κB)-dependent pDC responses such as expression of the differentiation markers CD40, CCR7, CD86, and tumor necrosis factor (TNF)-related apoptosis-inducing ligand (TRAIL), and secretion of the proinflammatory cytokines TNF-α and interleukin 6 (IL-6).

## RESULTS

We demonstrate that exposure of pDCs to HCV-infected hepatoma cells surprisingly did not induce phosphorylation of NF-κB or cell surface expression of CD40, CCR7, CD86, and TRAIL, or secretion of TNF-α and IL-6. In contrast, CpG-A and CpG-B induced production of TNF-α and IL-6 in pDCs exposed to the HCV-infected hepatoma cells, showing that cell-associated virus did not actively inhibit toll-like receptor (TLR)-mediated NF-κB phosphorylation.

## Conclusions

Our results suggest that cell associated HCV signals in pDCs via endocytosis-dependent mechanism and IRF7 but not via NF-κB pathway. In spite of IFN-α induction, cell-associated HCV does not induce a full functional response of pDCs. These findings contribute to the understanding of evasion of immune responses by HCV.

